# 20. Cost-Effectiveness of Recombinant Zoster Vaccine for Vaccinating Immunocompromised Adults Against Herpes Zoster in the United States

**DOI:** 10.1093/ofid/ofab466.222

**Published:** 2021-12-04

**Authors:** Desmond Curran, Ahmed Salem, Stéphane Lorenc, Brandon Patterson, Justin Carrico, Katherine A Hicks, Elizabeth M La, Sara Poston, Christopher F Carpenter

**Affiliations:** 1 GSK, Wavre, Vlaams-Brabant, Belgium; 2 Freelance Consultant, Wavre, Brabant Wallon, Belgium; 3 Former GSK, Current Janssen Global Services, Raritan, New Jersey; 4 RTI Health Solutions, Research Triangle Park, North Carolina; 5 Beaumont Hospital, Royal Oak, Michigan

## Abstract

**Background:**

Individuals who are immunocompromised (IC) due to disease or therapy are at increased risk of herpes zoster (HZ), with HZ cases in IC populations also resulting in increased health care resource use and costs as compared with the immunocompetent population. This study assesses the cost-effectiveness of recombinant zoster vaccine (RZV) versus no vaccine for the prevention of HZ in IC adults aged ≥ 18 years in the United States (US).

**Methods:**

A Markov model with a one-year cycle length was developed to follow a hypothetical cohort of one million IC individuals for a 30-year time horizon. The model estimates health and cost outcomes associated with RZV versus no vaccine. The base-case analysis considered hematopoietic stem cell transplant (HSCT) recipients who were assumed to remain IC for five years post-transplant. Second-dose compliance was assumed to be 100%, with efficacy and waning inputs based on clinical trial data. Epidemiological, cost, and utility inputs were obtained from standard US sources and published literature. Costs and quality-adjusted life-years (QALYs) were discounted at 3% per year. Sensitivity, threshold, and scenario analyses were conducted, including scenarios of four other IC conditions.

**Results:**

In the modeled hypothetical cohort of one million HSCT recipients, RZV resulted in 116,790 fewer HZ cases and 21,446 fewer postherpetic neuralgia cases versus no vaccine, 5,545 fewer QALYs lost and a societal cost-savings of &5.4 million. The number needed to vaccinate to prevent one HZ case was estimated to be 9. HSCT population results were shown to be robust in sensitivity and threshold analyses. In scenario analyses, RZV was cost saving for renal transplant recipients. Incremental cost-effectiveness ratios for other IC populations were &33,268 per QALY gained for human immunodeficiency virus, &67,682 for breast cancer, and &95,972 for Hodgkin lymphoma.

**Conclusion:**

Results suggest that RZV is a cost-effective option for vaccinating US IC adults for the prevention of HZ and associated complications.

**Disclosures:**

**Desmond Curran, PhD**, **The GSK group of companies** (Employee, Shareholder) **Ahmed Salem, MSc**, **The GSK group of companies** (Employee) **Stéphane Lorenc, NA**, **GSK group of companies** (Consultant) **Brandon Patterson, PharmD, PhD**, **GSK group of companies** (Shareholder) **Justin Carrico, BS**, **GSK group of companies** (Consultant)**RTI Health Solutions** (Employee) **Katherine A. Hicks, MS, BSPH**, **GSK group of companies** (Consultant)**RTI Health Solutions** (Employee) **Elizabeth M. La, PhD**, **The GSK group of companies** (Employee, Shareholder) **Sara Poston, PharmD**, **The GSK group of companies** (Employee, Shareholder) **Christopher F. Carpenter, MD, MHSA**, **GSK group of companies** (Consultant)

